# Ultrasound-Guided Transversus Abdominis Plane (TAP) Blocks Vs. Local Infiltration Anesthesia on Post Anesthesia Care Unit (PACU) Pain Control

**DOI:** 10.1093/asjof/ojad027.018

**Published:** 2023-04-14

**Authors:** Salomon Puyana, Jadyn Heffern, H Harvak Hajebian, Kevin Kresofsky, Abigail Chaffin, John Lindsey

**Affiliations:** Tulane University, New Orleans, LA; Tulane University School of Medicine, New Orleans, LA; Gartner Health Medical Center, Middletown; Tulane University School of Medicine, New Orleans, LA; Tulane University School of Medicine, New Orleans, LA; Tulane University School of Medicine, New Orleans, LA

## Abstract

**Goals/Purpose:**

Ultrasound-guided regional field blocks are not widely used in outpatient plastic surgery.^1^ These blocks have been shown to significantly decrease the number of prescribed opioids in patients who underwent abdominoplasty, liposuction, and primary submuscular breast operations. ^2,3^ The goal of this study is to compare ultrasound-guided TAP blocks to local infiltration anesthesia with or without blind rectus sheath blocks in patients undergoing abdominoplasty.

**Methods/Technique:**

A retrospective review was conducted of patients undergoing outpatient abdominoplasty performed by the senior surgeon. Group 1 (Local) received local infiltration anesthesia with or without blind rectus sheath blocks between April 2009 and December 2013. Group 2 (TAP) received surgeon-led, intraoperative, ultrasound-guided, 4-quadrant TAP blocks between January 2014 and December 2021. Outcomes measured included opioid utilization, morphine milligram equivalents (MME) and pain level at discharge (scale from 1 to 10).

All abdominoplasties were performed using the same suture materials and techniques by the senior surgeon in the same accredited outpatient surgical facility. Abdominoplasty technique included supraumbilical undermining, umbilical transposition, rectus abdominis fascial plication, progressive tension sutures, and liposuction of the flanks. Patients were excluded if other procedures were performed at the time of abdominoplasty.

**Results/Complications:**

60 patients in each of the two study groups met the study criteria for a total of 120 patients. The study groups were similar except for a lower average age in group 1 (Table 1). Patients receiving TAP blocks (group 2) had significantly lower MME requirements in the PACU (3.48 v 2.21, p=0.005). There was no difference in pain level at discharge between the two groups (2.35 v 2.17, p=0.624), (Table 1).

**Conclusion:**

Surgeon-led, intraoperative, ultrasound-guided, 4-quadrant TAP blocks statistically significantly reduced opioid utilization by 36.5% in the PACU while achieving comparable patient pain scores.

**Table 1.** Local vs. TAP Block Demographics and Outcomes

	Abdominoplasty		
	Local	TAP	*P*-value
**No. patients**	60	60	-
**Age (yrs)**	40.03 (23 - 74)	44.1 (25 - 74)	0.038
**Weight (lbs)**	161.96 (110 - 219)	165.62 (101 - 212)	0.446
**BMI (kg/m^2^)**	27.15 (20 - 36.46)	28.55 (17.59 - 39.29)	0.064
**Smoker**	0	0	-
**Diabetic**	1	0	-
**Pain level at discharge (0–10)**	2.35 (0 - 6)	2.17 (0 - 8)	0.609
**Morphine Milligram Equivalents (MME)**	3.48 (0 - 10)	2.21 (0 - 8)	0.005
